# New tricks for old targets: Anti-CTLA-4 antibodies re-envisioned for cancer immunotherapy

**DOI:** 10.18632/oncotarget.25800

**Published:** 2018-07-27

**Authors:** Jeremy D. Waight, Dhan Chand, David A. Savitsky

**Affiliations:** Agenus Inc., Lexington, MA, USA

**Keywords:** CTLA-4, Fcγ receptors, antibody engineering, TCR, cancer immunotherapy

Cytotoxic T-lymphocyte-associated protein 4 (CTLA-4) has emerged as an effective target for cancer immunotherapy [1]. As a potent negative regulator of T cell priming and immune cell activation, James Allison and colleagues hypothesized that blockade of CTLA-4 may enhance antitumor T cell activity in cancer patients by “releasing the brakes” on the immune system [1]. The approval of ipilimumab (Yervoy^®^, Bristol Myers-Squibb) for unresectable or metastatic melanoma in 2011 has validated this hypothesis. Nevertheless, restriction of durable antitumor responses to only a subset of patients has motivated us and others to better understand CTLA-4 biology and the mechanisms by which anti-CTLA-4 antibodies mediate their antitumor effects [2].

An early view for how anti-CTLA-4 antibodies increased immune responses was through liberation of B7 (CD80/86) from CTLA-4, ultimately permitting co-stimulation through B7-CD28 and activation of naïve T cells [1]. Antagonism of CTLA-4-mediated lipid raft disruption, trans-endocytosis of, and reverse signaling into B7 have since been described as potential mechanisms of action [3]. In parallel, an evolving understanding of Fc gamma receptor (FcγR) interactions and their importance for the activity of a range of immunotherapeutic antibodies has led to the discovery that antibody Fc-FcγR co-engagement is critically important for the antitumor effects of CTLA-4 antibodies [4, 5]. In preclinical mouse models, this Fc-dependent antitumor activity has been correlated with the depletion of intratumoral regulatory T cells (Tregs). However, we found that while treatment of tumor-bearing mice with anti-CTLA-4 antibody *(i)* results in profound tumor control and *(ii)* requires Fc-FcγR co-engagement, intratumoral Treg cell depletion is often incomplete [6]. Therefore, we asked whether the Fc-dependent activity of CTLA-4 antibodies is exclusively dependent on Treg cell depletion or whether the story is more nuanced.

Using a tumor-free mouse system (staphylococcal enterotoxin B administration), we interrogated the dependence of anti-CTLA-4 Fc-FcγR co-engagement on antigen-specific T cell activity *in vivo*. Consistent with observations in tumor-bearing mice, we found enhanced antigen-specific T cell responses to anti-CTLA-4 therapy could be controlled by simply modifying the Fc backbone of the antibody or lost following blockade of activating FcγRIV (CD16-2). Remarkably, we observed that responses elicited by anti-CTLA-4 were maintained in the absence of Treg cells. Our results implicated an unappreciated Fc-dependent mechanism employed by anti-CTLA-4 antibodies to enhance T cell responsiveness independent of Treg cell depletion.

**Figure 1:**
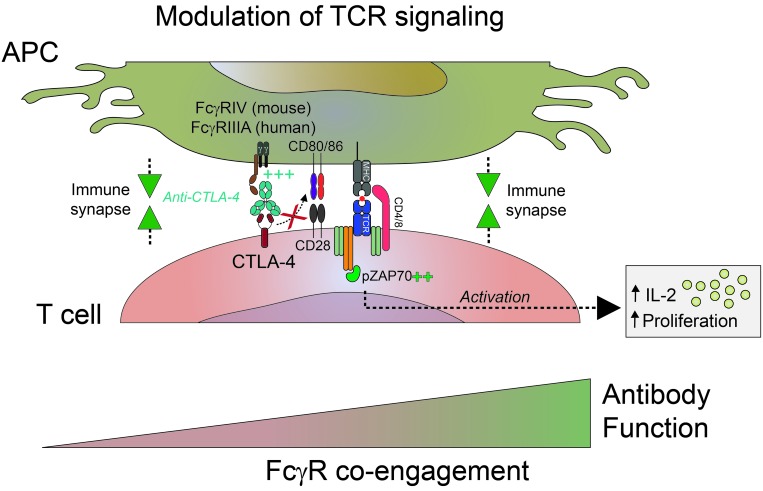
Optimizing Fc-FcγR co-engagement enhances the activity of CTLA-4 antagonist antibodies CTLA-4 antibodies with increased binding affinities to activating Fcγ receptors FcγRIV (mouse) or FcγRIIIA (human) augment T cell priming by improving the quality of the immune synapse between a T cell and an antigen presenting cell (APC). For additional details see [6].

A central aspect of our study was the concordance in observations between murine and human cells. In particular, we showed that FcγRIIIA (CD16) engagement was required for promoting anti-CTLA-4 activity, again highlighting the importance of activating FcγRs in facilitating antibody function. As an orthogonal approach, we generated several anti-CTLA-4 Fc variants and observed a robust correlation between affinity to FcγRIIIA and functional activity. This improved functional activity was associated with enhanced apical T cell receptor (TCR) signaling events, as determined by ZAP70 phosphorylation. Our data also suggest that this enhanced activity is T cell intrinsic and unlikely to be due to antigen presenting cell (APC) conditioning *via* reverse signaling into FcγRIIIA. We hypothesized that anti-CTLA-4 Fc-FcγRIIIA co-engagement helps “bridge” T cells and APCs to improve immune synapse quality and promote more effective T cell priming, an attribute that can be modulated by Fc engineering (Figure 1). This hypothesis aligns well with the model of kinetic segregation in which physical exclusion of phosphatases through tight T cell:APC interactions facilitates effective TCR signaling events [7]. Future studies are needed to fully characterize the effects of anti-CTLA-4 Fc-FcγR co-engagement on immune synapse quality.

Beyond CTLA-4, we demonstrated that antibodies targeting the checkpoint receptor T cell Immunoreceptor with Ig and ITIM domains (TIGIT) followed similar rules of FcγR engagement (*viz* improved Treg cell-independent activity with improved FcγRIIIA affinity). Interestingly, both TIGIT and CTLA-4 are direct regulators of TCR signaling and akin to anti-CTLA-4, TIGIT blocking antibodies that engage FcγRIIIA promote T cell responses *via* higher quality synapse formation and/or better co-stimulatory signal redirection. Given the relevance of TIGIT to NK cell biology, the question of how antibody Fc engineering will impact antitumor activity mediated by non-T cells may also be of interest [8].

Our work underscores the importance of FcγR co-engagement for anti-CTLA-4 therapy and is further supported by recent work from Arce Vargas and colleagues who demonstrated a striking correlation between FcγR biology and improved clinical response to ipilimumab in melanoma patients with high tumor mutational burden [9]. They noted that patients with the germ-line high affinity FcγRIIIA polymorphism (V158) exhibited a significant advantage in survival over those that harbored only the low-affinity FcγRIIIA allele (F158). Given these data, and ongoing clinical trials with modified anti-CTLA-4 variants (*e.g.* NCT03110107), it will be exciting to see if considerations for FcγR biology yield improved responses in patients. Of particular interest is whether anti-CTLA-4 variants engineered to enhance FcγRIIIA binding can generate meaningful responses, *via* improved T cell stimulation and Treg cell depletion, in patients that express the low-affinity FcγRIIIA. Finally, while the functional effects of modulating antibody Fc-FcγR co-engagement will vary depending on the target of interest, an overall understanding of the biology elicited by different antibody Fc formats is important to ensure that optimal therapies are progressed towards the clinic [10].
